# Strong Association between Inotrope Administration and Intraventricular Hemorrhage, Gestational Age, and the Use of Fentanyl in Very Low Gestational Age Infants: A Retrospective Study

**DOI:** 10.3390/children10101667

**Published:** 2023-10-08

**Authors:** Theodora Stathopoulou, Eleni Agakidou, Christos Paschaloudis, Angeliki Kontou, Ilias Chatzioannidis, Kosmas Sarafidis

**Affiliations:** Department of Neonatology and Neonatal Intensive Care, Faculty of Medicine, School of Health Sciences, Aristotle University of Thessaloniki, Ippokrateion General Hospital, 54642 Thessaloniki, Greece; dorastatho@gmail.com (T.S.); chrispashaloudis@hotmail.com (C.P.); angiekon2001@yahoo.gr (A.K.); ihatz@auth.gr (I.C.); kosaraf@auth.gr (K.S.)

**Keywords:** neonates, very low birth weight infants, clinical pharmacology, drug safety, fentanyl, inotrope, dopamine, epinephrine, blood pressure, intraventricular hemorrhage

## Abstract

This was a single center, retrospective cohort study designed to evaluate the association between the administration of inotropes to hypotensive very low gestational age infants (VLGAI) and prenatal and neonatal risk factors. Inpatient medical records were reviewed to identify neonates treated with inotropes (treated group) and a control group for comparison. Two hundred and twenty two (222) VLGAI (less than 32 weeks’ gestation) were included in the final analysis and were stratified based on timing of treatment with 83 infants (37.4%) and 139 infants (62.6%) in the treated and control groups, respectively. A total of 56/83 (67%) received inotropes for arterial hypotension during the first 3 days (early treatment subgroup) and 27/83 (32.5%) after 3 days of life (late-treated subgroup). Fentanyl, severe intraventricular hemorrhage (IVH), and gestational age (GA) were the risk factors most significantly associated with the need for inotrope use both during the first 3 days of life and the whole NICU stay, before and after adjustment for confounders. In conclusion, fentanyl, severe IVH, and GA are the risk factors most strongly associated with the need for inotrope treatment in VLGAI. Measures to modify these risk factors may decrease the need for cardiovascular medications and improve outcomes.

## 1. Introduction 

During the first weeks of life, the cardiovascular system of preterm neonates undergoes dramatic changes and hemodynamic adaptations potentially leading to arterial hypotension (AH). Therefore, AH is a frequent problem in very low gestational age infants (VLGAI), and is reported to occur in up to 20% of this vulnerable population in the first 48 h of life [1]. Relevant studies demonstrate that arterial pressure (AP) steadily increases over the first hours and days in VLGAI, suggesting that AH observed soon after birth may reflect a developmental phenomenon rather than a pathological entity that requires intervention. It has even been characterized as idiopathic or permissive hypotension. Previous authors, however, demonstrated that certain prenatal/perinatal factors may be associated with the development AH in VLGAI [2]. In addition, AH has been linked to severe acute complications of prematurity, such as peri-intraventricular hemorrhage (IVH), respiratory distress syndrome (RDS), sepsis, necrotizing enterocolitis (NEC), and increased mortality, as well as severe long-term morbidities and poor neurodevelopmental outcomes in survivors [3,4,5,6,7]. 

The importance of an early and prompt diagnosis along with appropriate management of AH in VLGAI is outlined by data from the Epipage 2 study. In this study, extremely preterm infants with isolated AH who did not receive any anti-hypotensive treatment were more likely to develop a brain injury compared to those who were treated for isolated hypotension [4]. On the other hand, treatment with inotropes has been identified as a significant risk factor independently associated with increased mortality and neurodevelopmental impairment [8,9]. This could justify the slight decrease in the use of inotropes observed overtime (from 2009–2013 to 2014–2015) [10]. Nevertheless, irrespective of the existing controversy surrounding the definition of AH in neonates, and the lack of randomized clinical trials (RCTs) [11], inotropes are still given in a considerable proportion of preterm infants. Published data from the US and Norway show that the rate of inotrope administration to NICU patients ranges between 2.7% and 25%, depending on gestational age (GA) [12,13]. 

In addition to prenatal and neonatal factors that may potentially affect the hemodynamic status of the neonate, it is clinically helpful to consider the timing after birth (early, within the first few days of life, versus late, beyond this time-period) where each predisposing factor may affect arterial blood pressure. This distinction may provide important insight into the underlying pathophysiology, and provide guidance towards the most appropriate therapeutic intervention. The primary aim of this study was to evaluate the association between the receipt of inotropes in hypotensive VLGAI during either the first 3 days of life or beyond this time-period, with prenatal and neonatal risk factors.

## 2. Patients and Methods

This is a single-center, retrospective, observational study involving a cohort of preterm infants with GA less than 32 weeks born between 2017 and 2022. Exclusion criteria included known severe congenital anomalies and infants for whom there was lack of adequate data for analysis. Medical records were reviewed to retrieve demographic, maternal, and neonatal data. Infants treated with inotropes for AH were designated as the treated group, while those who did not receive inotropes were designated as the control group. The decision to start anti-hypotensive treatment was at the discretion of the attending neonatologist, which was based on departmental protocols. In our center, published blood pressure thresholds [14] or the POCUSNEO free application (https://neopeds.academy/bp/ accessed on 15 November 2016) were used to define AH. Receipt of inotropes within the first three days of life was defined as early treatment, while therapy beginning beyond this day was defined as late treatment. Treatment for hypotension was defined as the use of monotherapy or a combination of anti-hypotensive agents including vasoactive medications (dopamine, dobutamine, epinephrine, norepinephrine, and milrinone) and cortisone. For the current study, the anti-hypotensive medications are collectively referred to as inotropes, since all neonates of the treated group received at least one inotrope/vasopressor medication combined or not with other vasoactive agents or cortisone. In our center, fentanyl is the first line analgesic used in infants undergoing tracheal intubation and/or receiving mechanical ventilation. Moreover, depending on the severity of pain, fentanyl is also given for postoperative analgesia alone or combined with paracetamol. Sedatives were administered to agitated infants with or without pain. IVH was classified according to the classification suggested by Papile et al. [15]. IVH of grades III and IV and periventricular leukomalacia were classified as severe peri-intraventricular hemorrhage (severe IVH). Definitions of the various perinatal and neonatal morbidities evaluated can be found with the Supplementary Material. 

The study protocol was reviewed and approved by the scientific committee of our hospital (Ref. number 37517/22 August 2023). All work was conducted in accordance with the declaration of Helsinki of 1975 [https://www.wma.net/what-we-do/medical-ethics/declaration-of-helsinki/, accessed on 10 January 2017], which was revised in 2013 and completed in 2016 by the Declaration of Taipei on Ethical Considerations regarding Health Databases and Biobanks (https://www.wma.net/what-we-do/medical-ethics/declaration-of-helsinki/, accessed on 10 January 2017). For purposes of personal data protection, each patient was referred to with an identification code, while names and other demographic data were deleted.

### Statistical Analysis

Continuous variables were presented as medians and upper/lower quartiles, since values of almost all of them did not follow the Gaussian distribution (Kolmogorov–Smirnov test). Categorical variables were presented as counts and proportions. Bivariate comparisons between the two groups were performed using the Mann–Whitney U test or the Kruskal–Wallis ANOVA for continuous variables and the Fisher’s exact test for categorical variables. A simple logistic regression model was fitted for every possible risk factor. Factors with *p* < 0.05 were included in a stepwise logistic regression model to assess the potential independent association of the clinical factors and interventions with the dependent variable (treatment with inotropes) after controlling for confounders. Log-likelihood test and the Akaike information criterion (AIC) values were used for comparison between models and selection of variables included in final model. The area under the ROC curve (AUC) with 95% confidence intervals was used to calculate the predictive power of the model. The cut-off point was selected based on the best trade-off between specificity and sensitivity. We fitted one multiple logistic regression model for early inotrope start and one for the treatment with inotropes during the whole stay in the neonatal intensive care unit (NICU). While fitting models, missing values were imputed by mean for continuous variables and most frequent value for categorical variables. Statistical significance for all hypothesis tests was set at *p* < 0.05. All statistical analyses were performed using the statistical computing language R, version 4.2.2, and the packages stats, MASS, pROC, and SPSS v.23. 

## 3. Results

**Figure 1:**
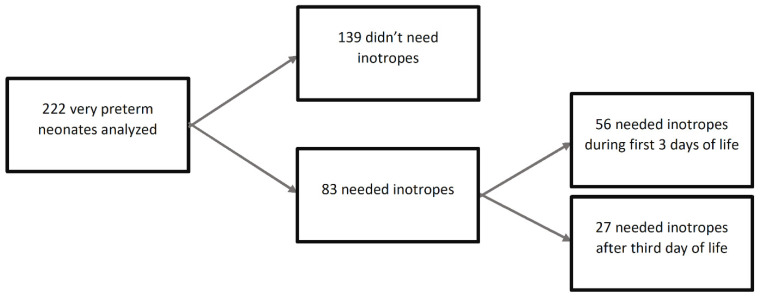
Flow chart of the studied population.

**Figure 2:**
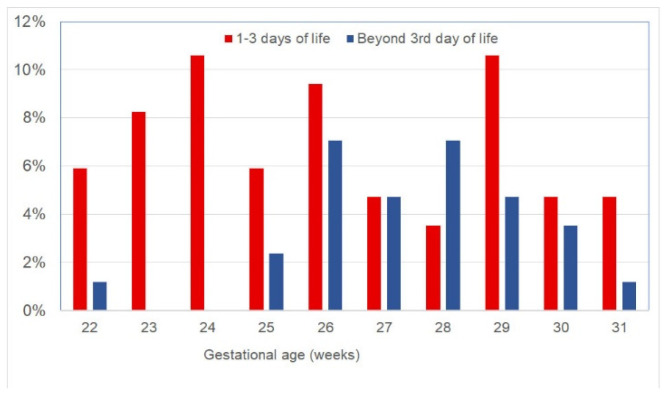
Proportion of very low gestational age infants in whom inotrope treatment was initiated either within the first three days of life or beyond this age, in relation to gestational age.

Data from 222 VLGAI were included in the final analysis. Of these, 83 (37.4%) infants received inotropes (treated group) and 139 (62.6%) did not receive inotropes (control group) (Figure 1). Out of the 83 infants in the treated group, 56 (67%) received support with inotropes within the first three days of life (early treatment subgroup) and 27 (33%) received inotropes beyond this age (late-treated subgroup). The inotropes administered to the study population are summarized in Table 1. Cortisone was given to four hypotensive extremely low birth weight (BW) neonates who did not respond to treatment with two inotropes. The proportion of VLGAI who received inotropes in relation to GA is shown in Figure 2. The GA (in weeks)-related distribution of the treated and control groups is shown in Supplementary Table S1. Moreover, the proportion of treated VLGAI in each week of GA is shown in Supplementary Table S2. 

### 3.1. Maternal, Prenatal, and Neonatal Characteristics of the Treated and Control Groups 

The demographic, prenatal, and neonatal data of the VLGAI that received inotropes at any time during the NICU stay and the control group are shown in Table 2. Compared to the control group, the treated group had significantly lower GA, BW, 1 min and 5 min Apgar scores, higher frequency of intubation and surfactant administration in the delivery room, and higher base deficit on admission to the NICU. In addition, the treated group was more likely to develop RDS of any grade, symptomatic patent ductus arteriosus (PDA), severe IVH, pulmonary hemorrhage, air-leak syndromes, sepsis (either early or late), and septic shock, and require mechanical ventilation. A higher proportion of the treated group received fentanyl, either as monotherapy or combined with other analgesics or sedatives. Remifentanil was given to only three infants in the present study, while other fentanyl derivatives or morphine were not used at all. The sedative and frequency of which they were administered to the study population were as follows: Phenobarbital (n = 25; 11.3%), ketamine (n = 14; 6.4%), midazolam (n = 6; 2.7%), propofol (n = 6; 2.7%), levetiracetam (Keppra; n = 6; 2.7%), and topiramate (n = 2; 0.9%). The incidence of NEC stage II–III, surgical NEC, and bronchopulmonary dysplasia of any grade did not differ significantly between the two groups. Compared to the controls, infants in the treated group had lower survival rates, while survivors stayed in the NICU for longer periods (Table 2).

### 3.2. Demographic, Prenatal, and Neonatal Data in the Early Treatment and Late-Treated Subgroups 

Comparison of demographic and prenatal–neonatal data between the early treatment and late-treated subgroups are summarized in Table 3. The early treatment subgroup had significantly lower GA, 5 min Apgar score, hematocrit, and incidence of intrauterine growth retardation, as well as lower pH and higher base deficit on admission to the NICU. In addition, the early treatment subgroup of infants were more likely to develop severe IVH and air-leak syndromes, and need non-invasive mechanical ventilation. The incidence of sepsis and septic shock were lower in the early treatment group (Table 3). 

### 3.3. Multiple Regression Analyses

The GA and BW were highly correlated (0.78, CI: 0.72–0.83, *p*-value < 0.001) with each other. Consequently, the logistic regression models could not include both variables at the same time due to multicollinearity. Therefore, in the regression models, we included only the GA, which was the criterion for recruitment. In addition, the number of infants studied did not allow the inclusion in regression analysis of all factors. 

**Figure 3:**
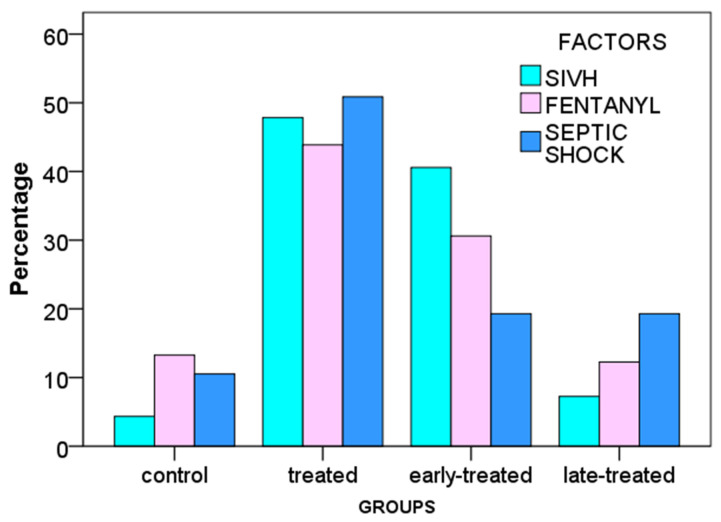
Proportion of very low gestational age infants with the risk factors most strongly associated with the inotrope treatment. SIVH, severe intra-periventricular hemorrhage.

Table 4 shows the selected risk factors, odds ratios with 95% confidence intervals, and *p*-values for inotropes commenced at any time during the NICU stay. The analysis reveals that after controlling for confounders, fentanyl administration and GA show the strongest independent association with the need for inotrope treatment (positive and inverse association, respectively) with odds ratio (OR) 4.661 (95% confidence intervals [95% CI] 2.047; 10.877; *p* < 0.001) for fentanyl use and OR 0.742 (95% CI 0.634; 0.863; *p* < 0.001) for GA. In addition, septic shock and severe IVH are also significant independent factors positively associated with the need for inotropes. The AUC value of the model is 0.883 with 95% CI 0.835–0.930. The optimal cut-off point is at 33.6%, with a specificity of 0.863 and sensitivity of 0.819. Confounders that were included in the stepwise regression but were not used in the final model were the 1 min Apgar score, delayed cord clamping, intubation and surfactant administration in the delivery room, base deficit on NICU admission, RDS, symptomatic PDA, pulmonary hemorrhage, air-leak syndromes, and application of mechanical ventilation (Table 4). Figure 3 shows the percentage of neonates having the risk factors most strongly associated with inotrope treatment.

Table 5 shows the logistic regression model results after taking into consideration the commencement of inotropes during the first three days of life. After adjustment for GA and other confounders, fentanyl administration and severe IVH were the risk factors having the strongest independent association with the need of inotrope treatment; OR 4.089; 95% CI 1.809; 9.329; *p* = 0.001 for fentanyl use and OR 5.544; 95% CI 2.032; 16.157; *p* = 0.001 for severe IVH. Additional significant independent factors were the GA (OR 0.769, 95% CI 0.648; 0.908) and base deficit on admission to the NICU (OR 0.900, 95% CI 0.806; 0.989). The AUC of the model is 0. 877 (95% CI 0.824; 0.932) and the optimal cut-off point is at 27.2% with a specificity of 0.861 and sensitivity of 0.804. Risk factors included in the stepwise regression that were not used in the final model were 1 min Apgar score, chorioamnionitis, delayed cord clamping, intubation and surfactant administration at birth, symptomatic PDA, pulmonary hemorrhage, and air-leak syndromes (Table 5).

## 4. Discussion

In the present study, bivariate comparisons between VLGAI treated with inotropes and controls show that several prenatal and neonatal factors differ significantly between the two groups. However, adjustment for GA and other significant confounders reveal that fentanyl administration, severe IVH, and GA are the risk factors most significantly associated with the need for inotrope support regardless of the time the AH developed. 

IVH is a serious complication of prematurity with severe acute and long-term consequences, depending on its severity. Predisposing factors in preterm infants include immaturity of autoregulation mechanisms and resultant fluctuations of cerebral blood flow secondary to variations in MAP, and partial pressure of carbon dioxide. Several studies in the late 1980s and early 1990s reported on the detrimental effect of AH on the development of IVH [16,17,18]. More recent studies also documented an association between AH and an increased risk of IVH before and after adjustment for potential confounders and regardless of the use of inotropes [6]. Kim et al., in a cohort of 166 infants with BW less than 1500 g (57% developed hypotension) also demonstrated that AH within the first week of life was significantly associated with severe IVH (higher than grade II), periventricular leukomalacia, and significantly lower scores of Bayley Scales of Infant and Toddler Development III [3].

On the other hand, additional data strongly indicate an association between the use of inotropes in preterm infants and severe IVH and adverse neurodevelopmental outcomes. In a retrospective study involving extremely low BW infants, treatment of AH within the first 72 h of life was associated with severe IVH (grade III–IV), longer hospitalization, and death. Surviving infants who received inotropes were more likely to have delayed motor development and hearing loss [19]. Likewise, Batton et al., in a prospective observational study of 367 infants 23^+0/7^ to 26^+6/7^ weeks’ GA evaluating the potential effect of anti-hypotensive treatment on clinical outcomes found that anti-hypotensive treatment was associated with an increased incidence of severe IVH (22% vs. 11%, *p* < 0.01) and mortality rate (67% vs. 78%, *p* = 0.02), regardless of the definition of hypotension [20]. Similar findings were reported by Vesoulis et al., who showed that the exposure to inotropes was significantly higher in preterm infants with severe IVH compared to those without severe IVH [21]. In contrast, in a randomized study of 98 preterm infants with hypotension and 70 controls, although a significantly higher rate of severe IVH was found in the hypotensive versus the control group, multiple regression analysis did not show any association between IVH and the use of inotropes/vasopressor drugs. Moreover, no difference could be documented regarding neurodevelopmental outcome and death up to the second to third year of life [22]. Other studies have also failed to document any significant association of early hypotension treated with anti-hypotensive agents with neurodevelopmental outcomes of very low BW infants [23,24,25].

Despite the published data, there remains controversy as to whether IVH and adverse neurodevelopmental outcomes are causally linked to inotrope treatment, the preexisting hypotension, or the low GA. Our results confirm a significant association of the inotrope administration with severe IVH, albeit due to the retrospective design, our study cannot support a cause–effect relationship between the inotrope treatment and IVH [26]. 

Numerous previous studies reported that low GA and BW are significant predictors of hypotension and the use of anti-hypotensive medications in preterm infants. Early studies by Zubrow et al. (1995) and Hegyi et al. (1994) found that GA and BW were strongly associated with AP within the first day of life, which progressively increased with postconceptional age [27,28]. More recently, several authors reported that infants who received anti-hypotensive treatment had lower GA and BW and increased clinical severity score. No other antenatal/perinatal factor was significantly associated with AH and treatment with anti-hypotensive medications in very preterm infants after adjustment for confounders [6,29,30,31]. Several studies confirmed the positive association of AP in preterm infants with the GA and/or BW, which were the strongest factors influencing neonatal AP [2,29,32,33,34]. In line with these reports, we found an inverse correlation between the GA and the prevalence of AH as well as treatment with vasoactive medications, regardless of the age at treatment initiation. 

Opioids, especially fentanyl, are the analgesics most commonly administered to prevent or treat neonatal pain [35]. This is also the case in our NICU, where fentanyl is the most often used opioid for procedural or post-surgery pain, either as monotherapy or in combination with other analgesics and/or sedatives. However, the potential adverse effects of fentanyl on the developing brain secondary to its hypotensive effect is a major source of concern, especially considering the immaturity of cerebral autoregulation in preterm infants, with little ability to compensate for circulation fluctuations [36]. However, there are considerable gaps of knowledge regarding the use of fentanyl in preterm infants. Three RCTs failed to demonstrate any significant effect of fentanyl on AP [37,38,39]. Several systematic reviews evaluating the pharmacological management of neonatal pain in various situations also failed to clarify the association of fentanyl with AH in preterm infants [40,41,42,43,44]. The general conclusion was that the evidence for fentanyl use outside of “*select clinical scenarios*” is limited [42]. Alternative analgesics with less effect on AP are being suggested but their analgesic effect and safety still need to be confirmed in well-designed clinical studies [42,45]. In this context, the significant association of inotrope use with fentanyl administration found in our study should be cautiously interpreted. It may simply reflect the increased incidence of painful/stressful complications and interventions in VLGAI, such as RDS, PDA, sepsis, NEC, and mechanical ventilation. These factors are more common in VLGAI and may cause both hypotension and/or pain or stress needing analgesia. It should be noted though that the significant independent association of inotrope treatment with fentanyl remained even after adjustment for GA and other confounders potentially associated with hypotension. Therefore, this issue needs to be clarified in further well-designed RCTs, recruiting adequate numbers of preterm infants, a task that presents serious ethical and technical issues.

On bivariate comparisons, we found significant differences in the incidence of several morbidities between the treated and control groups (shown in Table 1). This could be attributed to the lower GA in treated infants, a major factor predisposed to the complications of prematurity. These, in turn, can adversely affect the hemodynamic status of the VLGAI leading to AH and inotrope support. Whether GA *per se* has an independent effect on the development of hypotension could only be answered in the context of a case–control study of non-treated and treated infants of comparable GA. In the present study, this was not possible due to the inadequate number of normotensive subjects born at less than 26 weeks’ gestation (see Supplementary Table S1). Moreover, multiple regression analysis shows that beyond GA, additional risk factors such as IVH, fentanyl administration, and septic shock are independently associated with the use of inotropes, even after adjustment for GA and other confounders. Regarding early and late initiation of inotropic support, infants treated early had significantly lower GA and were more clinically ill at birth (as indicated by the 5 min Apgar score, base deficit, pH on NICU admission, and the incidence of IUGR). These differences could provide a plausible explanation for the need of early intervention. On the contrary, as sepsis and septic shock usually develop beyond the third postnatal day, and BPD well beyond birth, treatment of AH with inotropes may be needed later during the NICU stay. 

Our study is the first to report on the strong independent association of fentanyl administration with the use of inotropes, an issue for which the published literature is inconclusive. Should our results be confirmed by future clinical trials, they may considerably limit the use of fentanyl in clinical practice and favor the use of analgesics/sedatives with minimal effect on AP, as well as the use of non-pharmacological means to mitigate neonatal pain and/or stress of VLGAI. The use of GA instead of BW as criterion of recruitment excluded more mature SGA infants with BW less than 1500 g. However, our study has limitations that should be taken into consideration. Due to the retrospective design, the present study may be influenced by bias related to the neonatologist’s decision to commence anti-hypotensive treatment, although the protocols of the department guided the decision. Moreover, Due to the retrospective design, our study cannot determine a cause–effect relationship between the inotrope treatment and risk factors. Specifically, our results cannot discriminate whether the severe IVH (which could potentially cause hypotension), or GA, or fentanyl, or other risk factors, which are found to be significantly independently related to the need for inotropic therapy, are associated with the need for inotrope treatment in a cause-and-effect relationship, or if are they simply confounding factors. Of note, a cause–effect relationship can be determined only using prospective studies [26]. Finally, our findings may only apply in VLGAI who share the characteristics of our study population. 

In conclusion, in our study, several important clinical factors associated with the use of inotrope agents were identified, including the strong association of fentanyl administration to VLGAI subjected to stressful/painful procedures and surgeries. In the context of the existing uncertainty, the strong association between fentanyl administration and treatment with inotropes documented in this study is an alarming finding that needs further evaluation. Other factors strongly associated with the use of inotropes were the severe IVH and GA. Measures to modify these risk factors, mostly prevention of IVH and reasonable use of fentanyl, may decrease the need for inotropic medications and improve outcomes. 

## Figures and Tables

**Table 1:** Frequency of inotrope administration to the VLGAI of the treatment group.

Inotrope	Number (%) of Treated Neonates
Dopamine	73 (88)
Dobutamine	37 (44.6)
Epinephrine	13 (15.7)
Norepinephrine	11 (13.3)
Milrinone	5 (6.0)

VLGAI, very low gestational age infants.

**Table 2:** Maternal perinatal and neonatal data of the treated and control groups.

Characteristics	Groups of Neonates		*p*-Value
	Control	Treated	
N	139	83	
**Maternal data**			
Hypertension ^$^	25 (18.0)	12 (14.5)	0.751 ^£^
Chorioamnionitis ^$^	17 (12.2)	17 (20.5)	0.123 ^£^
Prenatal steroids ^$^	123 (88.5)	74 (89.2)	1.0 ^£^
Mg administration ^$^	60 (46.5)	40 (51.9)	0.541 ^£^
Anesthesia for labor/CS ^$^	117 (84.2)	60 (72.3)	0.176 ^£^
Caesarean section ^$^	122 (87.8)	65 (78.3)	0.093 ^£^
Inborn ^$^	133 (95.7)	77 (92.8)	0.534 ^£^
Delayed cord clamping ^$^	70 (50.4)	30 (36.7)	0.055 ^£^
PPROM ^$^	36 (25.9)	26 (31.3)	0.473 ^£^
**Neonatal data**			
Gestational age (wks) *	30 (28; 31)	26 (24; 29)	<0.001 ^#^
Birth weight (g) *	1270 (1110; 1540)	810 (632; 1085)	<0.001 ^#^
1 min Apgar score *	7 (6; 8)	5 (4; 7)	<0.001 ^#^
5 min Apgar score *	9 (8; 9)	8 (7; 8)	<0.001 ^#^
Base deficit on NICU admission *	−5.4 (−7.3; −3.6)	−7 (−9.6; −4.8)	<0.001 ^#^
pH on admission *	7.32 (7.27; 7.38)	7.30 (7.24; 7.37)	0.101 ^#^
Male sex ^$^	55 (39.6)	43 (51.8)	0.102 ^£^
SGA ^$^	10 (7.2)	10 (12.0)	0.327 ^£^
IUGR ^$^	23 (16.5)	17 (20.5)	0.557 ^£^
Intubation at birth ^$^	26 (18.7)	52 (62.7)	<0.001 ^£^
Surfactant at birth ^$^	14 (10.1)	27 (32.5)	<0.001 ^£^
Symptomatic PDA ^$^	13 (9.4)	35 (42.2)	<0.001 ^£^
Severe IVH ^$^	3 (2.2)	33 (39.8)	<0.001 ^£^
Pulmonary hemorrhage ^$^	8 (5.8)	21 (25.3)	<0.001 ^£^
Air-leak syndromes ^$^	5 (3.6)	9 (10.8)	0.044 ^£^
Sepsis (confirmed early or late) ^$^	50 (36)	49 (59)	0.001 ^£^
Septic shock ^$^	6 (4.3)	28 (33.7)	<0.001 ^£^
Fentanyl monotherapy ^$^	11 (7.9)	21 (25.3)	0.002 ^£^
Sedative monotherapy ^$^	0 (0)	10 (12)	<0.001 ^£^
Fentanyl and sedatives ^$^	2 (1.4)	22 (26.5)	<0.001 ^£^
NEC stage II, III ^$^	8 (5.8)	10 (12.0)	0.159 ^£^
Surgical NEC ^$^	2 (1.4)	6 (7.2)	0.055 ^£^
BPD any grade ^$^	52 (37.4)	27 (32.5)	0.555 ^£^
RDS ^$^	73 (52.5)	62 (74.7)	0.001 ^£^
Overall mechanical ventilation ^$^	125 (89.9)	82 (98.8)	0.011 ^£^
Invasive ventilation ^$^	46 (33.1)	80 (96.4)	<0.001 ^£^
Non-invasive ventilation ^$^	116 (83.5)	42 (50.6)	<0.001 ^£^
Survival ^$^	130 (96.3)	34 (42.0)	<0.001 ^£^
Days in NICU for all population *	50 (41; 69.7)	17 (6.3; 80.5)	<0.001 ^#^
Days in NICU for survivors *	52 (42; 70)	95 (63; 134)	<0.001 ^#^
Days in NICU for non-survivors *	5.0 (1.5; 12.5)	7.5 (2.5; 15)	0.432 ^#^

* Median (quartile 1; quartile 3); ^$^ counts (%); ^#^ Mann–Whitney test; ^£^ Fisher’s exact test; BPD, bronchopulmonary dysplasia; CS, caesarean section; IUGR, intrauterine growth restriction; IVH, peri-intraventricular hemorrhage; NEC, necrotizing enterocolitis; NICU, neonatal intensive care unit; Mg, magnesium; PDA, patent ductus arteriosus; PPROM, preterm premature rupture of membranes; RDS, respiratory distress syndrome; SGA, small for gestational age; wks, weeks.

**Table 3:** Maternal perinatal and neonatal data of the early- and late-treated subgroups.

Characteristics	Subgroups of Treated Neonates		*p*
	Early-Treated	Late -Treated	
N	56	27	
**Maternal data**			
Hypertension ^$^	5 (10)	7 (28)	0.091 ^£^
Chorioamnionitis ^$^	14 (26.9)	3 (11.1)	0.150 ^£^
Prenatal steroids ^$^	49 (87.5)	25 (92.6)	0.711 ^£^
Mg ^$^	25 (48.1)	15 (60.0)	0.461 ^£^
Anesthesia for labor/CS ^$^	37 (66.2)	23 (85.2)	0.140 ^£^
Caesarean section ^$^	43 (76.8)	22 (81.5)	0.779 ^£^
Inborn ^$^	53 (94.6)	24 (88.9)	0.385 ^£^
Delayed cord clamping ^$^	16 (28.6)	14 (51.9)	0.068 ^£^
PPROM ^$^	20 (35.7)	6 (22.2)	0.323 ^£^
**Neonatal data**			
Gestational age (wks) *	26 (24; 29)	27 (24; 27)	0.018 ^#^
Birth weight (g) *	725 (602; 1220)	865 (746; 1220)	0.194 ^#^
1 min Apgar score *	5 (3; 7)	7 (4; 7)	0.092 ^#^
5 min Apgar score *	8 (7; 8)	8 (8; 9)	0.001 ^#^
Base deficit on NICU admission *	−7.9 (−12.6; −6.0)	−5 (−6.6; −3.2)	<0.001 ^#^
pH on admission *	7.29 (7.23; 7.35)	7.33 (7.30; 7.41)	0.008 ^#^
Male sex ^$^	31 (55.4)	12 (44.4)	0.064 ^£^
SGA ^$^	4 (7.1)	6 (22.2)	0.071 ^£^
IUGR ^$^	7 (12.5)	10 (37.0)	0.021 ^£^
Intubation at birth ^$^	39 (69.6)	13 (48.1)	0.098 ^£^
Surfactant at birth ^$^	19 (33.9)	8 (29.6)	0.805 ^£^
Symptomatic PDA ^$^	23 (41.1)	12/27 (44.4)	0.957 ^£^
Severe IVH ^$^	28 (50.0)	5 (18.5)	0.008 ^£^
Pulmonary hemorrhage ^$^	16 (28.6)	5 (18.5)	0.423 ^£^
Air-leak syndromes ^$^	9 (16.1)	0 (0)	0.028 ^£^
Sepsis (confirmed early or late) ^$^	28 (50.0)	21 (77.8)	0.030 ^£^
Septic shock ^$^	13 (23.2)	15 (55.6)	0.008 ^£^
Fentanyl monotherapy ^$^	13 (23.2)	7 (25.9)	1.0 ^£^
Sedative monotherapy ^$^	6 (10.7)	4 (15.4)	0.718 ^£^
Fentanyl and sedatives ^$^	17 (30.4)	5 (18.5)	0.423 ^£^
NEC stage II–III ^$^	4 (7.2)	6 (22.2)	0.071 ^£^
Surgical NEC ^$^	3 (5.4)	3 (11.1)	0.385 ^£^
BPD any stage ^$^	13 (23.2)	14 (51.9)	0.018 ^£^
RDS ^$^	40 (71.4)	22 (81.5)	0.423 ^£^
Overall mechanical ventilation ^$^	55 (98.2)	27 (100)	0.999 ^£^
Invasive ventilation ^$^	54 (96.4)	26 (96.3)	0.999 ^£^
Non-invasive ventilation ^$^	21 (37.5)	21 (13.3)	0.001 ^£^
Survival ^$^	17 (30.9)	17 (65.4)	0.004 ^£^
Days in NICU for all population *	11 (4; 50)	63 (23.5; 98.5)	<0.001 ^#^
Days in NICU for survivors *	88 (62; 133)	65 (63; 138)	0.823 ^#^
Days in NICU for non-survivors *	7 (1.7; 12.5)	16.5 (6.75; 28.5)	0.014 ^#^

* Median (quartile 1; quartile 3); ^$^ counts (%); ^#^ Mann–Whitney test; ^£^ Fisher’s exact test; BPD, bronchopulmonary dysplasia; CS, caesarean section; IUGR, intrauterine growth restriction; IVH, peri-intraventricular hemorrhage; NEC, necrotizing enterocolitis; NICU, neonatal intensive care unit; Mg, magnesium; PDA, patent ductus arteriosus; PPROM, preterm premature rupture of membranes; RDS, respiratory distress syndrome; SGA, small for gestational age; wks, weeks.

**Table 4:** Stepwise regression analysis and discriminating ability; significant risk factors independently associated with inotropes commenced at any time during NICU stay (dependent variable).

Significant Independent Factors	Odds Ratio	Confidence Intervals		*p*-Value
		Lower	Upper	
GA	0.74	0.63	0.86	<0.001
Fentanyl	4.66	2.05	10.88	<0.001
SIVH	6.67	1.94	31.19	0.01
Septic shock	4.95	1.76	15.45	<0.001
**Discriminating ability**				
AUC	0.88	0.84	0.93	
Threshold	0.34			
Specificity	0.86			
Sensitivity	0.82			

AUC, area under the curve; GA, gestational age; SIVH, severe peri-intraventricular hemorrhage. Risk factors included in the stepwise regression that were not used in the final model were the 1 min Apgar score, delayed cord clamping, intubation and surfactant administration in the delivery room, base deficit on NICU admission, respiratory distress syndrome, symptomatic patent ductus arteriosus, pulmonary hemorrhage, air-leak syndromes, mechanical ventilation.

**Table 5:** Logistic, stepwise regression analysis, and discriminate accuracy of the model; significant risk factors independently associated with inotrope treatment commenced within the first three days of life (dependent variable).

Significant Independent Factors	Odds Ratio	Confidence Intervals		*p*-Value
		Lower	Upper	
GA	0.769	0.648	0.908	0.002
Base deficit	0.900	0.806	0.989	0.042
Fentanyl ± sedatives	4.089	1.809	9.329	0.001
SIVH	5.544	2.032	16.157	0.001
**Discriminating ability**				
AUC	0.877	0.824	0.932	
Threshold	0.272			
Specificity	0.861			
Sensitivity	0.804			

AUC, area under the curve; GA, gestational age; SIVH, severe peri-intraventricular hemorrhage. Risk factors included in the stepwise regression that were not used in the final model were 1 min Apgar score, chorioamnionitis, delayed cord clamping, intubation and surfactant administration at birth, symptomatic patent ductus arteriosus, pulmonary hemorrhage, and air-leak syndromes.

## Data Availability

The data presented in this study are available on request from the corresponding author.

## Supplementary Materials

The following supporting information can be downloaded at: https://www.mdpi.com/article/10.3390/children10101667/s1, Definitions of the neonatal complications used in this study. Table S1. GA (in weeks)—related distribution of the study population in the control and treated groups. Table S2. Numbers and proportions of VLGAI who received inotropes in each week of GA.
