# Supplementary material for: Strong Association between Inotrope Administration and Intraventricular Hemorrhage, Gestational Age, and the Use of Fentanyl in Very Low Gestational Age Infants: A Retrospective Study
Source: Children (Basel). 2023 Oct 8;10(10):1667. doi: 10.3390/children10101667 (PMC10605532; doi:10.3390/children10101667)
Supplement: Supplementary file 1 [file children-10-01667-s001.zip › children-2599169-supplementary.pdf]

SUPPLEMENTARY MATERIAL

Patients and methods.

Definitions used in this study.

Gestational age was determined on the basis of prenatal ultrasonography and/or the date of the last menstrual period. If the best obstetrical estimate was unavailable, determination of gestational age was based on the neonatologist's estimate with the use of the New Ballard Score.

Small for gestational age (SGA) was defined as birth weight below the 10th percentile on the Fenton preterm growth charts.

Intrauterine growth restriction (IUGR) diagnosis was based on prenatal growth rate and placenta size assessed by ultrasound.

Neonates with positive blood cultures for microbes or fungi were considered as having proven **sepsis**, whereas those with clinical and laboratory evidence of sepsis but negative blood cultures as having possible sepsis.

Necrotizing enterocolitis (NEC) was defined as Bell's stage II to III, according to the modified Bell's classification. **Surgical NEC** included cases requiring surgical management of the disease.

Respiratory distress syndrome (RDS) was considered in infants who developed respiratory distress soon after birth with lung X-ray findings compatible with pulmonary immaturity and/or surfactant deficiency.

Bronchopulmonary dysplasia (BPD) was defined as the need for supplemental oxygen at 36 weeks of postmenstrual age.

Diagnosis of **symptomatic patent ductus arteriosus** (PDA) was based on clinical (murmur, hyperdynamic precordium, and bounding pulses) and echocardiography findings (left-to-right shunting, left heart dimensions, and left atrium to aorta ratio).

Chorioamnionitis was defined either as intrapartum fever (>37.8ºC) along with one or more additional clinical/laboratory findings, such as maternal and fetal tachycardia, foul-smelling amniotic fluid, and leukocytosis (clinical chorioamnionitis), or as histologically confirmed chorioamnionitis.

Supplementary Table S1. GA (in weeks) - related distribution of the study population in the control and treated groups

GA (weeks)	Control group (n= 139)		Treated group (n= 83)	
	N	%	N	%
22	1	0.7	6	7.2
23	1	0.7	7	8.4
24	2	1.4	11	13.3
25	2	1.4	8	9.6
26	9	6.5	14	16.9
27	3	2.1	8	16.9
28	22	15.8	8	16.1
29	20	13.4	11	13.3
30	28	20.1	5	6.0
31	51	36.0	5	6.0

Supplementary table S2. Numbers and proportions of VLGAI who received inotropes in each week of GA.

GA (weeks)	Total population of VLGAI	Treated VLGAI	Proportion of treated VLGAI
	N	N	%
22	7	6	85.7
23	8	7	87.5
24	13	11	84.6
25	10	8	80.0
26	23	14	60.9
27	11	8	72.7
28	30	8	26.7
29	31	11	34.5
30	33	5	15.2
31	56	5	8.9
TOTAL	222	83	37.4

GA, gestational age; VLGAI, very low gestational age infants
